# COVID-19 vaccine uptake among health care workers in Ghana: a case for targeted vaccine deployment campaigns in the global south

**DOI:** 10.1186/s12960-021-00657-1

**Published:** 2021-11-06

**Authors:** Robert Kaba Alhassan, Seth Owusu-Agyei, Evelyn Korkor Ansah, Margaret Gyapong

**Affiliations:** Institute of Health Research (IHR), University of Health and Allied Sciences, PMB 31, Volta Region, Ho, Ghana

**Keywords:** Coronavirus disease 2019 (COVID-19), Vaccine, Health care workers, Trial, Willingness, Uptake, Ghana

## Abstract

**Background:**

Health care workers (HCWs) are among the high-risk groups in contracting and dying from COVID-19. World Health Organization estimates that over 10,000 HCWs in Africa have been infected with COVID-19 making it a significant occupational health hazard to HCWs. In Ghana, over 100 HCWs have already been infected and dozen others died from the virus. Acceptability and uptake of the COVID-19 vaccine is therefore critical to promote health and safety of HCWs as the country battles out of a third wave of the pandemic.

**Objective:**

The study sought to ascertain the correlates of HCWs likelihood of participating in a COVID-19 vaccine trial and accepting the vaccine when given the opportunity.

**Methods:**

The study was a web-based cross-sectional survey among HCWs (*n* = 1605) in all sixteen (16) administrative regions in Ghana. Data were analyzed with STATA statistical analysis software (version 14). Chi-square (*X*^2^) and Fisher’s exact tests were used to test for differences in categorical variables; bivariate probit regression analysis with Average Marginal Effect (AME) was employed to ascertain the determinants of HCWs’ likelihood of participating in a COVID-19 vaccine trial and taking the vaccine.

**Results:**

It was found that 48% of HCWs will participate in a COVID-19 vaccine trial when given the opportunity; 70% will accept the COVID-19 vaccine; younger HCWs (AME = 0.28, SE = 0.16, *p* < 0.1), non-Christians (AME = 21, SE = 0.09, *p* < 0.05) and those who worked in faith-based health facilities (AME = 18, SE = 0.07, *p* < 0.05) were more likely to participate in a COVID-19 vaccine trial. Female HCWs (AME = − 11, SE = 0.04, *p* < 0.05) and those with lower educational qualification were less likely to accept a COVID-19 vaccine (AME = − 0.16, SE = 0.08, *p* < 0.1). Reasons cited for unwillingness to participate in a COVID-19 vaccine trial or uptake the vaccine were mainly fear, safety concerns, mistrust, uncertainty, spiritual and religious beliefs.

**Conclusions:**

Acceptance of the COVID-19 vaccine appear to be high among HCWs; conversely, willingness to volunteer for the vaccine trial was low. Continuous targeted and integrated public health education for HCWs will enhance vaccine acceptability to promote safety and population health in the global south as Ghana intensifies efforts to produce COVID-19 vaccines locally.

**Supplementary Information:**

The online version contains supplementary material available at 10.1186/s12960-021-00657-1.

## Background

As at August, 2021, over 200 million cases of the novel coronavirus disease 2019 (COVID-19) were recorded globally. Out of this figure over 4 million people unfortunately died [1]. Within the African region over 4 million cases have been recorded so far with over 100 000 deaths [2]. In Ghana, more than 100 000 cases of COVID-19 have been recorded with over 1000 deaths [3].

Public health response across the globe at the initial phase of the pandemic generally focused on non-pharmaceutical interventions (NPIs) such as frequent hand washing, physical/social distancing and the use of face masks in public spaces [2]. Albeit these NPIs remain essential in the fight against the global pandemic, effective vaccine deployment is an ultimate intervention to propel humanity back to normal lives. In the later part of 2020, breakthroughs were announced of different variants of COVID-19 vaccines which got World Health Organization (WHO) emergency use authorization (EUA) [4].

At the time of writing this paper, at least seven different COVID-19 vaccines on three platforms have been approved by the WHO [4] and over billion doses of the vaccine already administered globally as at 4th May, 2021. Nonetheless, this figure represents barely 7.9% of the global population receiving first dose and another 3.6% receiving second dose of the vaccine [4].

Africa has so far administered first dose of the COVID-19 vaccine to barely 0.99% of its population and second dose administered to 0.35% of the continent’s population as at May, 2021 [2]. Health care workers (HCWs) are among the high-risk groups in terms of contracting the virus and equally dying from it because of their closer contact with COVID-19 cases [5].

WHO African Regional Office estimates that over 10 000 HCWs in Africa have been infected with the disease [6, 7]. In Ghana, anecdotal information suggests over 100 HCWs have already been infected and over 10 others died from the virus. To address this occupational health hazard, HCWs are among the prioritized category of persons earmarked for vaccination against the virus to promote their safety. Even though the vaccine does not give absolute protection from infection, available empirical data suggests it reduces risk of hospitalization and other severe outcomes including death [8–11].

Nonetheless, there are concerns of fear, anxiety and hesitancy among the populace including HCWs which threatens successful rollout of the vaccine in many countries including Ghana. Ghana like many African countries has had its first share of resistance to vaccine trials on account of fear, anxiety, lack of trust and conspiracy theories. In the year 2015, there was suspension of a planned Phase 1 trial for the Janssen Ebola vaccine at the University of Health and Allied Sciences (UHAS) campus in Hohoe, Volta region in Ghana, and Phase 2 trial for the GlaxoSmithKline (GSK) Ebola vaccine in Hohoe and Kintampo also in Ghana [12]. Similarly, in 2003 in northern Nigeria, polio vaccination campaigns were boycotted [13] largely because of earlier experience related to an antibiotic trial (Trovan) in 1996 when 11 children were reported to have died, even though there was no evidence the deaths were due to the trial [13]. Recent malaria vaccine equally met some resistance at the early stages in Ghana which nearly halted the trial but was finally rolled out on 30th April 2019 [14].

Even though vaccine hesitancy, even among HCWs, remains an important threat to successful rollout of vaccines there are still limited scientific studies within the Ghanaian context to unearth the correlates of the phenomenon. Moreover, few studies have investigated into HCWs likelihood of accepting to participating in COVID-19 vaccine trial and uptake of the vaccine, when given the opportunity. Available studies, on COVID-19 vaccine uptake are either outside the Ghanaian context or conducted after deployment of the COVID-19 vaccine when citizens were already exposed to reactogenicity of the vaccine [15–22]. As Ghana intensifies in efforts towards producing the COVID-19 vaccines locally [23] and possibly conducting clinical trials on such vaccines, it is imperative the process is driven by empirical data on likelihood of stakeholders accepting to participate in these trials and uptake the vaccines.

This study therefore sought to examine the correlates of HCWs likelihood of participating in a COVID-19 vaccine trial and eventually uptake the vaccine when given the opportunity. The study was conducted between 18th September and 23rd October, 2020, before the first batch of COVAX facility of AstraZeneca vaccine was delivered in Ghana for the first time in the sub-region [24].

## Methods

### Study design

The study was a cross-sectional descriptive survey conducted prior to deployment of COVID-19 vaccine in Ghana. The study sought to ascertain frontline health workers’ likelihood of participating in a COVID-19 vaccine trial and uptake the vaccine when given the opportunity.

### Study setting/population/sampling

The study was conducted in all sixteen (16) administrative regions in Ghana targeting frontline HCWs at various levels of the healthcare delivery system. Ghana had over 115 000 HCWs employed in the public sector as at 2018, with nearly 60% being nurses and midwives [25], cited in Asamani et al. [26]. Unpublished data suggests the HCWs workforce has appreciated over the 2018 figure. Using Krejcie and Morgan’s [27] formula for calculating sample size based on known population, the minimum required sample size of 384 at 95% confidence level was deemed representative. Nonetheless, a total of 1605 responses were received from the nationwide web-based survey, representing at least 10% coverage of the population of health workforce in Ghana.

### Instruments of data collection

The study employed a web-based nationwide survey using RedCap software. Questionnaire administration was done via social media platforms and networks between 18th September and 23rd October, 2020 to eligible respondents who voluntarily consented to participate in the survey. Participants who could not access the web-based survey link were assisted to answer the questions by trained research assistants from all the 16 regions. The data collection instrument comprised of open and close ended questions. The questionnaire sections were on socio-demographic characteristics; experiences with COVID-19; views on COVID-19 vaccine trials and the vaccine, and likelihood of accepting vaccination when given the opportunity. Internal reliability of the questionnaire items was tested using Cronbach’s alpha test and the average scale reliability coefficient was above the 80% rule of thumb.

### Ethical considerations

The study got ethical clearance from the Research Ethics Committee (REC) of the University of Health and Allied Sciences, Ghana (Clearance Number: UHAS-REC A.1[6]20-21). Participation was voluntary and only participants who provided voluntary informed consent were included in the study. Privacy and confidentiality were assured through coding to anonymize personal information of respondents.

### Data analysis

Data were analyzed with STATA statistical analysis software (version 14) after cleaning and coding. Main outcome variables of interest were whether or not respondents will participate in a COVID-19 vaccine trial (yes = 1; no = 0) and accept or uptake a COVID-19 vaccine when given the opportunity (yes = 1; no = 0). Explanatory variables of interest were: sex (male = 1; female = 2); age (numeric); education (multiple response); professional category (multiple response); level of health facility where staff works (multiple response); region (multiple response); marital status (multiple response); religion (multiple response); willingness to pay for COVID-19 vaccine (yes = 1, no = 2) and whether or not staff works in a COVID-19 treatment centre (yes = 1, no = 2).

Chi-square (*X*^2^) and Fisher’s exact tests were used to test for differences in categorical variables at 95% confidence level. Additionally, bivariate probit regression analysis using Average Marginal Effect (AME) estimations was employed to ascertain the determinants of HCWs’ readiness to participate in a COVID-19 vaccine trial and their likelihood of taking the vaccine when given the opportunity. The use of the bivariate probit regression is justified because of the dichotomous nature of the dependent variables [28]. Also, since the bivariate probit regression is a nonlinear model, its coefficients do not sufficiently inform on the magnitude of the effects of changes in the explanatory variables on an outcome variable [29]. In view of these strengths, AMEs of the explanatory variables were used in order to make their interpretations intuitively meaningful. Moreover, AMEs instead of the Marginal Effects at the Means (MEMs) were used because the former is deemed to be superior to the latter [30].

All explanatory variables of interest were tested for multicollinearity before fitting them in the regression models and variables with Variance Inflation Factor (VIF) above the 10.0 rule of thumb were dropped. Apart from age, all the remaining explanatory variables were treated as dummy variables because of their categorical nature. It must however be stressed that, North East and Savannah regions had one respondent each and Ahafo region had eight respondents. The few number of respondents led to multicollinearity; hence, the variable “region” was recoded to merge “North East” and “Savannah” regions to “Northern” region. Similarly, “Ahafo”, “Bono” and “Bono East” were merged and recorded as “Brong-Ahafo” region for same reasons. Doing so is justified because these regions were each one administrative region before the demarcation of Ghana’s new regions in 2019.

### Findings

#### Background information of respondents

Total of 1605 responses were received from the web-based survey. After data cleaning 1159 complete responses were retained representing 72% return rate. Approximately 62% of the respondents were females; the mean age was 31.91 ± 10. Majority of the respondents were married (54%) and Christian (92%); likewise, respondents from Greater Accra region (22%) dominated while respondents with at least diploma qualification constituted approximately 35%; 77% of the respondents worked in government-owned facilities with 34% of them working for 2–5 years; out of the 1095 respondents who answered the question on whether they worked in a COVID-19 treatment centre at the time of answering the questionnaire, 74% of them said no. Overall, over 90% of the 1126 respondents were clinical frontline staff and in terms of the professional category of the respondents, nurses dominated representing nearly 50% followed by midwives (18%). The least category was medical officers who constituted barely 2% of the study sample (see Table 1).

**Table 1:** Background characteristics of respondents

Variables	Statistics	
Sex of respondent	Freq	Percent
Male	436	38.18
Female	706	61.82
Total	1142	100.00
Age range of respondent		
18–23	50	4.66
24–29	302	28.15
30–35	503	46.88
36–41	168	15.66
42–47	31	2.89
48+	19	1.77
Total	1073	100.00
Marital status of respondent		
Divorced/widowed	17	1.54
Living together	19	1.72
Married	594	53.85
Never married	457	41.43
Separated	16	1.45
Total	1103	100.00
Religious affiliation of respondent		
Christian	1016	92.03
Moslem/Traditionalist/other	88	7.97
Total	1104	100.00
Region of residence of respondent		
Ashanti	61	5.77
Ahafo	9	0.85
Brong Ahafo	38	3.60
Bono East	45	4.26
Central	85	8.04
Eastern	98	9.27
Greater Accra	228	21.57
Northern/North East	63	5.96
Savannah	13	1.23
Upper East	60	5.68
Upper West	33	3.12
V olta/Oti	219	20.72
Western/Western North	105	9.93
Total	1057	100.00
Educational level of respondent		
Certificate	349	31.08
Diploma	391	34.82
Bachelors	335	29.83
Masters/PhD/fellowship	48	4.27
Total	1123	100.00
Student	**52**	**4.62**
Allied health	**186**	**16.52**
Medical officer	**20**	**1.78**
Midwife	**197**	**17.50**
Nurse	**560**	**49.73**
Pharmacist	**19**	**1.69**
Other health staff	**92**	**8.17**
Total	1126	100.00
Workplace of respondent		
Government	829	77.40
Private	71	6.63
Mission	113	10.55
Quasi/Other	58	5.42
Total	1071	100.00
Work experience of health staff		
1 year or less	238	22.60
2–5 years	358	34.00
6–9 years	289	27.45
10 years or more	168	15.95
Total	1053	100.00
Respondents working in COVID-19 treatment centre		
No	813	74.25
Yes	282	25.75
Total	1095	100.00
Participate in COVID-19 vaccine trial		
No	393	52.33
Yes	358	47.67
Total	751	100.00
Advice someone to participate in a COVID-19 vaccine trial		
No	354	47.64
Yes	389	52.36
Total	743	100.00
Accept to be immunized with a COVID-19 vaccine		
No	223	29.85
Yes	524	70.15
Total	747	100.00
Advice someone to be immunized with a COVID-19 vaccine		
No	184	24.76
Yes	559	75.24
Total	743	100.00

Bold values indicate occupation/professional category

Source: field Data (2020); Legend: COVID-19 (Coronavirus Disease 2019)

### Willingness to participate in COVID-19 vaccine trial and uptake the vaccine

It was observed that 48% of the respondents said they will participate in a COVID-19 vaccine trial when given the opportunity; 52% will advise someone to participate in the COVID-19 vaccine trial; 70% of the respondents indicated their willingness to accept the COVID-19 vaccine when given the opportunity, and 75% will advise someone to take the vaccine (see Table 1). Table 2 shows details of disaggregated responses on willingness to participate in a vaccine trial and accept the vaccine according to age, gender, religion, marital status, educational qualification, type of health facility and professional category.

**Table 2:** Cross-tabulations on willingness to participate in COVID-19 vaccine trial and uptake the vaccine

Variables	Accept COVID-19 vaccination			Participate in COVID-19 vaccine trial		
Sex	No	Yes	Total	No	Yes	Total
Male	75	232	307	144	165	309
Female	146	288	434	246	190	436
Total	221	520	741	390	355	745
Age range						
18–23	7	26	33	23	11	34
24–29	54	140	194	106	89	195
30–35	95	234	329	165	164	329
36–41	35	74	109	49	59	108
42–47	9	13	22	15	8	23
48 and above	5	6	11	8	4	12
Total	205	493	698	366	335	701
Marital status						
Divorced	3	4	7	4	3	7
Living together	6	10	16	11	5	16
Married	114	254	368	159	205	364
Never married	90	231	321	162	159	321
Separated	3	6	9	6	3	9
Widowed	0	2	2	1	1	2
Total	216	507	723	343	376	719
Educational level						
Certificate	63	154	217	107	112	219
Diploma	76	174	250	129	122	251
Bachelor degree	76	155	231	131	101	232
Master degree	6	21	27	13	14	27
Fellowship	0	2	2	1	1	2
PhD	0	3	3	0	3	3
Total	221	509	730	381	353	734
Professional category						
Allied health professional	29	84	113	45	67	112
Medical officer	3	14	17	6	11	17
Midwife	43	76	119	63	56	119
Nurse	116	260	376	175	196	371
Pharmacist	4	7	11	4	7	11
Student	6	28	34	17	17	34
Other	19	46	65	37	30	67
Total	220	515	735	347	384	731
Facility ownership						
Public/government	156	382	538	277	262	539
Private	25	30	55	32	24	56
Mission	16	52	68	26	42	68
Quasi-government	14	13	27	19	8	27
Other	6	5	11	10	1	11
Total	217	482	699	364	337	701
Health facility level						
Teaching hospital	23	44	67	42	26	68
Regional hospital	9	18	27	15	12	27
District hospital	83	176	259	152	107	259
Polyclinic/clinic	30	53	83	43	40	83
Health centre	47	117	164	67	98	165
CHPS compound	21	64	85	38	47	85
Total	213	472	685	357	330	687
Work in COVID-19 centre						
No	161	360	521	265	257	522
Yes	56	134	190	105	86	191
Total	217	494	711	370	343	713
Voluntarily test for COVID-19						
Very unlikely						
Unlikely	10	26	36	23	13	36
Undecided	22	28	50	34	17	51
Likely	26	39	65	44	23	67
Very likely	85	223	308	154	155	309
Total	79	206	285	137	148	285
Ever tested for COVID-19						
Yes	76	171	247	121	127	248
No	140	342	482	262	223	485
Prefer not to disclose	5	4	9	6	3	9
Total	221	517	738	389	353	742

Source: field Data (2020); Legend: CHPS (Community-based health planning and services)

### Determinants of voluntary participation in COVID-19 vaccine trial

Significant determinants of likelihood of participating in COVID-19 vaccine trial were: age, religious affiliation and ownership of health facility where the staff works. For instance, health workers aged 36–41 years were 28 times more likely to voluntarily participate in a COVID-19 vaccine trial (AME = 0.28, SE = 0.16, *p* < 0.1) than persons aged 18–23 years; persons aged 48 years or more were 38 times less likely to advise someone to participate in a COVID-19 vaccine trial (AME = −38, SE = 0.20, *p* < 0.1) relative to persons aged 18–23 years. Non-Christians were 21 more likely to voluntarily participate in a COVID-19 vaccine trial (AME = 21, SE = 0.09, *p* < 0.05) and recommend same to someone (AME = 19, SE = 0.09, *p* < 0.05) relative to Christians. Relative to health staff working in government-owned health facilities, those working in Mission health facilities were more likely to voluntarily participate in COVID-19 vaccine trial (AME = 18, SE = 0.07, *p* < 0.05) and recommend same to others (AME = 15, SE = 0.07, *p* < 0.05). Conversely, staff working in quasi-government facilities were 27 times less likely to participate in COVID-19 vaccine trial (AME = −27, SE = 0.08, *p* < 0.01) and recommend same to others (AME = −24, SE = 0.09, *p* < 0.01) compared to those in the government-owned facilities (see Table 3).

**Table 3:** Probit regression estimates of determinants of willingness to participate in COVID-19 vaccine trial

	Self	Someone
Sex (Ref: male)		
Female	−0.04 (0.05)	−0.05 (0.05)
Age (Ref: 18–23)		
24–29	0.22 (0.15)	−0.01 (0.14)
30–35	0.16 (0.15)	−0.05 (0.15)
36–41	0.28^*^ (0.16)	0.04 (0.16)
42–47	0.07 (0.19)	−0.19 (0.19)
48+	−0.11 (0.20)	−0.38^*^ (0.20)
Marital status (Ref: divorced/widowed)		
Living together	−0.24 (0.23)	−0.30 (0.23)
Married	0.06 (0.19)	0.06 (0.20)
Never married	−0.002 (0.20)	0.01 (0.20)
Separated	−0.13 (0.28)	−0.17 (0.28)
Region (Ref: Ashanti)		
Ahafo	0.03 (0.26)	−0.00 (0.26)
Brong Ahafo	0.11 (0.15)	0.18 (0.15)
Bono East	0.08 (0.15)	0.09 (0.15)
Central	−0.03 (0.11)	0.01 (0.11)
Eastern	−0.01 (0.11)	0.02 (0.11)
Greater Accra	0.02 (0.10)	−0.01 (0.10)
Northern/North East	0.05 (0.12)	0.01 (0.12)
Savannah	−0.18 (0.17)	0.06 (0.21)
Upper East	0.18 (0.12)	0.13 (0.13)
Upper West	−0.00 (0.15)	0.03 (0.15)
Volta/Oti	−0.02 (0.10)	0.03 (0.10)
Western/Western North	0.08 (0.11)	0.06 (0.11)
Religion (Christian)		
Moslem/traditionalist/other	0.21^**^ (0.09)	0.19^**^ (0.09)
Education (Ref: certificate)		
Diploma	0.00 (0.06)	0.02 (0.06)
Bachelors	−0.08 (0.06)	−0.04 (0.06)
Masters/PhD/fellowship	0.06 (0.12)	0.06 (0.12)
Professional category (Ref: student)		
Allied health	0.15 (0.10)	0.03 (0.10)
Medical officer	0.11 (0.15)	0.04 (0.15)
Midwife	−0.02 (0.10)	−0.09 (0.10)
Nurse	0.10 (0.09)	−0.02 (0.09)
Pharmacist	−0.09 (0.17)	0.01 (0.19)
Physician assistant	0.04 (0.18)	0.05 (0.18)
Workplace (Ref: public)		
Private	0.03 (0.08)	−0.03 (0.08)
Mission	0.18^***^ (0.07)	0.15^**^ (0.07)
Quasi/Other	−0.27^***^ (0.08)	−0.24^***^ (0.09)
Experience (1 year or less)		
2–5 years	0.05 (0.06)	0.05 (0.06)
6–9 years	0.06 (0.07)	0.04 (0.07)
10 years or more	0.12 (0.08)	0.12 (0.09)
COVID-19 treatment centre (Ref: no)		
Yes	−0.06 (0.05)	−0.07 (0.05)
Observations	569	563

Average Marginal Effects; standard errors in parentheses; ^*^ *p* < 0.1, ^**^ *p* < 0.05, ^***^ *p* < 0.01

### Correlates of staff willingness to uptake COVID-19 vaccine

The results further revealed that females were 11 times less likely to accept a COVID-19 vaccine when given the opportunity compared to males (AME = −11, SE = 0.04, *p* < 0.05); females were also 10 times less likely to advise someone to take the vaccine (AME = −10, SE = 0.04, *p* < 0.05) than their male counterparts. Older respondents (48 years and above) were 35 times less likely to recommend a COVID-19 vaccine to someone (AME = −35, SE = 0.21, *p* < 0.1) relative to younger age group (18–23 years).

Similarly, persons whose marital status was “living together” were 33 times less likely to recommend a COVID-19 vaccine to someone relative to divorced/widowed respondents (AME = −33, SE = 0.20, *p* < 0.1). Non-Christians were 20 times more likely to accept a COVID-19 vaccine (AME = 0.20, SE = 0.06, *p* < 0.01) and 20 times more likely to recommend same to others (AME = 0.17, SE = 0.06, *p* < 0.01) than Christians. Persons who had higher education (i.e., Masters/PhD/fellowship qualification) were 16 times more likely to accept a COVID-19 vaccine (AME = 0.16, SE = 0.08, *p* < 0.1) and 19 times more likely recommend same to others (AME = 0.19, SE = 0.07, *p* < 0.01) as compared to those with lower qualification. Staff who worked in private health facilities were 17 times less likely to participate in a COVID-19 vaccine trial (AME = −0.17, SE = 0.08, *p* < 0.05) and 12 times less likely to recommend same to others (AME = −0.12, SE = 0.07, *p* < 0.1) relative to those working in government-owned facilities. Finally, respondents who worked in quasi-government facilities were 34 less likely to accept a COVID-19 vaccine (AME = −34, SE = 0.09, *p* < 0.01) and 31 times less likely to recommend such a vaccine to others (AME = −31, SE = 0.10, *p* < 0.01) as compared to staff working in government-owned facilities (see Table 4).

**Table 4:** Probit regression estimates of determinants of willingness to uptake COVID-19 vaccine

	Self	Someone
Sex (Ref: male)		
Female	−0.11^**^ (0.04)	−0.10^**^ (0.04)
Age (Ref: 18–23)		
24–29	0.03 (0.12)	0.02 (0.11)
30–35	−0.02 (0.12)	−0.00 (0.12)
36–41	−0.07 (0.14)	0.03 (0.13)
42–47	−0.12 (0.18)	−0.15 (0.18)
48+	−0.30 (0.21)	−0.35^*^ (0.21)
Marital status (ref: divorced/widowed)		
Living together	−0.29 (0.21)	−0.33^*^ (0.20)
Married	−0.10 (0.14)	−0.04 (0.13)
Never married	−0.11 (0.15)	−0.10 (0.14)
Separated	−0.08 (0.22)	−0.25 (0.23)
Region (Ref: Ashanti)		
Ahafo	0.06 (0.24)	0.08 (0.22)
Brong Ahafo	0.04 (0.15)	0.19 (0.13)
Bono East	−0.08 (0.15)	0.07 (0.14)
Central	0.02 (0.10)	0.09 (0.10)
Eastern	0.11 (0.10)	0.15 (0.10)
Greater Accra	0.05 (0.09)	0.09 (0.09)
Northern/North East	0.02 (0.12)	0.07 (0.11)
Savannah	−0.06 (0.24)	−0.04 (0.24)
Upper East	0.08 (0.12)	0.12 (0.12)
Upper West	−0.01 (0.15)	0.16 (0.13)
Volta/Oti	0.00 (0.09)	0.09 (0.09)
Western/Western North	0.13 (0.10)	0.12 (0.10)
Religion (Ref: Christian)		
Moslem/Traditionalist/other	0.20^***^ (0.06)	0.17^***^ (0.06)
Education (Ref: certificate)		
Diploma	−0.01 (0.05)	0.03 (0.05)
Bachelors	−0.01 (0.05)	0.03 (0.05)
Masters/PhD/fellowship	0.16^*^ (0.08)	0.19^***^ (0.07)
Professional category (Ref: student)		
Allied health	−0.00 (0.09)	0.05 (0.09)
Medical officer	0.07 (0.13)	0.09 (0.13)
Midwife	0.01 (0.09)	0.01 (0.09)
Nurse	−0.03 (0.08)	0.02 (0.08)
Pharmacist	−0.10 (0.18)	0.10 (0.14)
Physician assistant	0.08 (0.14)	0.14 (0.12)
Workplace (Ref: public)		
Private	−0.17^**^ (0.08)	−0.12^*^ (0.07)
Mission	0.10^*^ (0.06)	0.05 (0.05)
Quasi/other	−0.34^***^ (0.09)	−0.31^***^ (0.10)
Experience (Ref: 1 year or less)		
2–5 years	−0.07 (0.06)	−0.08 (0.05)
6–9 years	−0.02 (0.06)	−0.03 (0.06)
10 years or more	−0.00 (0.08)	−0.02 (0.07)
COVID-19 treatment centre (Ref: No)		
Yes	−0.01 (0.04)	0.01 (0.04)
Observations	568	565

Average Marginal Effects; standard errors in parentheses; ^*^ *p* < 0.1, ^**^ *p* < 0.05, ^***^ *p* < 0.01

Follow-up questions revealed that the predominant reasons cited by HCWs for their unwillingness to participate in a COVID-19 vaccine trial and uptake the vaccine included fear, health and safety concerns, mistrust, uncertainty, spiritual and religious beliefs (see Additional file 1 for details).

## Discussion

HCWs are a critical piece of every health system across the globe [31]. Outbreak of the COVID-19 pandemic and the devastating effect on healthcare delivery has re-emphasized the important role of HCWs in every health system [32]. Safety of HCWs is particularly central in the response of nations against the pandemic. While some countries have performed relatively better in terms of protecting these frontline critical staff, same cannot be said of the resource-poor countries in the global south like Ghana [7, 33, 34].

There are varied reports on the number of COVID-19 infections and deaths among frontline healthcare workers (HCW) [33]. For instance, WHO African Regional Office estimates over 10 000 healthcare workers in Africa were infected with the virus in 2020 [7]. Likewise, the WHO Pan American Regional Office (PAHO) reported that 570 000 HCWs were infected and 2500 died due to COVID-19 as at September, 2020 [34]. Even though there is paucity of empirical data on COVID-19 related deaths of HCWs in Ghana, available anecdotal reports suggest over 2000 HCWs have been infected and at least 6 deaths recorded [35]. Even though this study started months before deployment of the COVID-19 vaccine, the current literature suggests high mortality rates among HCWs due to COVID-19 is largely due to limited vaccination coverage and inadequate supply of personal protective equipment (PPEs) [33].

It is therefore not out of place that many countries globally have prioritized HCWs for vaccination against COVID-19 [15, 36–39]. Unfortunately, the all-time record breakthrough in development and deployment of the COVID-19 vaccine has induced scepticisms and safety concerns on the vaccine. These concerns have impacted negatively on acceptance of this important intervention to control and eventually eradicate the pandemic.

Ghana’s history on resistance to vaccine trials and vaccine uptake is not too different from many African countries. Key among the historical antecedents are the suspension of the Ebola vaccine Phase 1 trial in 2015 [12] and early resistance to the ongoing malaria vaccine trial (RTS,S) which eventually started in April, 2019 [13] after successful stakeholder engagements. Reviewed literature points to the huge mass media misinformation (i.e., linkage with 5G technology and so-called vaccine chip implantation in the human body) on the COVID-19 vaccine as a major barrier to acceptance and uptake. This misinformation is coupled with the unbridled negative influence by some religious leaders and groups on public opinion concerning vaccine trials and vaccine uptake including the COVID-19 vaccine [40].

Unfortunately, there is limited empirical evidence on sources of the skepticism and vaccine trial hesitancy, especially among HCWs. Available literature has largely concentrated on community perceptions and willingness to participate in the COVID-19 trials and subsequently, accept the vaccine. This study is therefore timely, because local pharmaceutical companies have intensified efforts to locally produce some of the COVID-19 vaccines in Ghana [23] and perspectives of HCWs is crucial to a successful rollout of this intervention.

As demonstrated in this study, willingness to participate in a COVID-19 vaccine trial was 48% compared to 70% in a similar study by Kitonsa et al. [41] among HCWs in Uganda. Likelihood of accepting the COVID-19 vaccine was however high (70%) suggesting low vaccine hesitancy among HCWs in Ghana. Feedback from the HCWs suggests Ghana is possibly among the few countries with a relatively high vaccine acceptance rate among HCWs in the sub-region relative to Nigeria [42], DRC [43] and outside the sub-region such as France [18].

It was observed that gender, age, religious affiliation and ownership of health facility where the staff works are significant correlates of HCWs’ likelihood of participating in a COVID-19 vaccine trial and accept the vaccine. Older staff and those of Christian religious faith were found to be more risk-averse as far as participation in the COVID-19 vaccine trial and uptake of the vaccine are concerned. Additionally, staff working in Mission facilities were more likely to participate in COVID-19 vaccine trial relative to government-owned facilities. This observation corroborates findings of similar studies which intimated personal and institutional level characteristics of HCWs potentially influence likelihood of accepting the COVID-19 vaccine trial and vaccine uptake [44, 45]. Future qualitative studies could help “dig deep” into reasons for these dynamics since the current study was a survey that did not explore the depth in terms of these views.

It was also found that females and older staff were more risk-averse as far as likelihood of taking the COVID-19 vaccine is concerned. Enitan et al. [38] found similar results in the case of Nigeria. Similarly, staff with relatively higher educational qualification were more likely to uptake the COVID-19 compared to those with lower educational qualifications which suggests limited exposure to information potentially entrenches misconceptions on the COVID-19 vaccine and hinders its uptake.

Staff working in private health facilities were less likely to accept the COVID-19 immunization compared to staff working in government-owned facilities. Further research is required to unearth reasons for these differentials in the various categories of health facilities, particularly in terms of type, level and the mix of staff found in these types of facilities to inform tailor made vaccine promotion campaigns among the different cadre of HCWs based on their peculiar needs.

An integrated yet multisectoral approach is imperative to ensure targeted vaccine promotion campaigns to address hesitancy among HCWs and the community at large as alluded to by Afolabi and Ilesanmi [40] and in the case of South Sudan [46]. Agyekum et al. [47] arrived at similar findings on vaccine hesitancy among health workers in Ghana, thus corroborating results of this study and the need for immediate interventions to further improve perceptions of HCWs since they are expected to be good ambassadors of this public health intervention to help control the pandemic.

Finally, concerns of fear, safety, mistrust and uncertainty intimated by the HCWs must be addressed through tailored messaging to promote goodwill and confidence in the vaccine as alluded to by Dror et al. [48]. Addressing concerns of HCWs on COVID-19 vaccine will help allay their fears and empower them as advocates to champion effective rollout of the vaccine to the general population.

## Conclusion

Likelihood of accepting the COVID-19 vaccine was found to be high among HCWs in Ghana albeit willingness to volunteer for the vaccine trial was relatively low. Nonetheless, this observation suggests Ghana, compared to Nigeria [42] and DRC [43], is doing better. A similar study in Uganda [41] revealed a vaccine acceptance rate of over 70%. The current findings on the acceptance rate demonstrates some level of confidence on the COVID-19 vaccine among HCWs in Ghana which needs to be sustained through continuous targeted public health education and sensitization seminars for HCWs. COVID-19 vaccine rollout interventions for the global south should therefore be guided by these country-specific context to promote effective implementation and success of these interventions.

Even though the findings reported in this paper are exploratory and might not entirely be conclusive, the results have adduced baseline empirical evidence on the need for more follow-up mixed methods studies to fully understand experiences and reasons for vaccine acceptance and hesitancy among HCWs. HCWs are an important source of health information and validation for the general public and their perspectives on the COVID-19 vaccine is critical to influencing public opinion on vaccinations in general.

Additionally, continuous engagement with HCWs by health managers and policy experts throughout the value chain on vaccine trials and deployment will promote trust and confidence which will likely trickle down to the general population through advocacy by HCWs on public health benefits of vaccination.

Finally, there is the need for effective stakeholder engagements towards championing vaccinations uptake as a public health intervention. Government agencies, Civil Society Organizations (CSOs), health professional groups should lead in mobilizing resources for these campaigns. The Ghana Medical Association (GMA), Ghana Registered Nurses and Midwives Association (GRNMA) and allied health professional groups should lead the crusade on educating their members as agents of change. Likewise, these health professional groups should prioritize scientific research to inform health advocacies and public education on the COVID-19 vaccine.

## Limitations

It is important to acknowledge that this survey was conducted several months before the deployment of the COVID-19 vaccine in Ghana. In light of this lag in time,  perspectives are likely to be different among the same study populations after some HCWs have now taken the vaccines and experienced reactogenicity of the vaccine. Moreover, the survey was web-based and might have exposed the design to selection bias since persons who owned a smartphone might have predominantly self-selected into the study. Nonetheless, use of research assistants to administer the questionnaire in-person to respondents who could not access the web-based survey minimized impact of the potential selection bias. Additionally, the nationwide scope with large sample size coupled with the use of pre-tested and validated tools render the findings valid and compelling.

### Implications for public health policy

While acknowledging the above limitations associated with this study, the following policy recommendations are proposed based on the study findings:

1. Health professionals’ regulatory councils in Ghana in collaboration with their employers and Health Facilities Regulatory Agency (HeFRA) should consider instituting mandatory vaccination for HCWs as a safety precaution. Section 22(1) of the Public Health Act, 2012 (Act 851) could be invoked for this purpose
2. The Ministry of Health (MoH) and Food and Drugs Authority (FDA) of Ghana should institute mandatory community-based stakeholder engagement protocols throughout the value chain of vaccine development, trials and rollout to enhance trust and acceptance in vaccines.
3. Health promotion unit of the Ghana Health Service (GHS) in collaboration with the information services department should collaborate to develop targeted national health information policy guidelines on vaccines to address the peculiar knowledge gaps of HCWs and the general population on vaccination.
4. Ministry of Health (MoH) and its implementing agents should collaborate with religious institutions and their leadership to identify and empower community champions to advocate for vaccine uptake.

## Supplementary Information

**Additional file 1: Table S1.** Verbatim reasons cited for unwillingness to participate in a COVID-19 vaccine Trial and uptake the vaccine.

## Data Availability

All data generated or analyzed during this study are included in this published article and its Additional file 1.

## Abbreviations

AME: Average Marginal Effects
AU: African Union
CDC: Centres for Disease Control and Prevention
COVID-19: Coronavirus Disease 2019
CSOs: Civil Society Organizations
DRC: Democratic Republic of Congo
EUA: Emergency use authorization
GHS: Ghana Health Service
GMA: Medical Association of Ghana
GRNMA: Ghana Registered Nurses and Midwives Association
GSK: GlaxoSmithKline
HCWs: Health Care Workers
MEMs: Marginal Effects at the Means
MoH: Ministry of Health
NPIs: Non-pharmaceutical interventions
PAHO: Pan American Regional Office
REC: Research Ethics Committee
SE: Standard error
UHAS: University of Health and Allied Sciences
VIF: Variance Inflation Factor
WHO: World Health Organization
