# Supplementary material for: COVID-19 vaccine uptake among health care workers in Ghana: a case for targeted vaccine deployment campaigns in the global south
Source: Hum Resour Health. 2021 Nov 6;19:136. doi: 10.1186/s12960-021-00657-1 (PMC8571849; doi:10.1186/s12960-021-00657-1)
Supplement: Supplementary file 1 — Additional file 1: Table S1. Verbatim reasons cited for unwillingness to participate in a COVID-19 vaccine Trial and uptake the vaccine. [file 12960_2021_657_MOESM1_ESM.rtf]

Table S1: Verbatim reasons cited for unwillingness to participate in a COVID-19 vaccine Trial and uptake the vaccine 
	Freq.	Percent	Cum.	
Because am allergic to some medication	1	0.70	0.70	
Above	1	0.70	1.41	
Afraid	1	0.70	2.11	
Afraid	1	0.70	2.82	
Am not interested	1	0.70	3.52	
Am scared	1	0.70	4.23	
As stated above	1	0.70	4.93	
Because I don't trust it	1	0.70	5.63	
Because i don't trust the vaccine	1	0.70	6.34	
Because is trail and error	1	0.70	7.04	
Because there is no approved vaccine	1	0.70	7.75	
Because there is no potent vaccine of covid 19	1	0.70	8.45	
Cause i don't trust it	1	0.70	9.15	
Cos I don't know what might be the outcome	1	0.70	9.86	
Cos am avoiding risk of some deformity	1	0.70	10.56	
Covid 19 is a fiasco	1	0.70	11.27	
Don't know how safe the vaccine is	1	0.70	11.97	
Don't know of side effects	1	0.70	12.68	
Don't trust it	1	0.70	13.38	
Dont have much information on vacvine to make informed decision	1	0.70	14.08	
Efficacy of vaccine not proven to be ??	1	0.70	14.79	
Fear	2	1.41	16.20	
Fear of getting wrong vaccination	1	0.70	16.90	
Fear of unknown  outcome	1	0.70	17.61	
Future complications	1	0.70	18.31	
Hypersensitivity to vaccines	1	0.70	19.01	
I am Afraid	1	0.70	19.72	
I believe in treatment with drugs.	1	0.70	20.42	
I do not take vaccine	1	0.70	21.13	
I don't  know the vaccine	1	0.70	21.83	
I don't have mich information about the vaccine	1	0.70	22.54	
I don't know if it is safe	1	0.70	23.24	
I don't know the safety of the vaccine	1	0.70	23.94	
I don't know the source	1	0.70	24.65	
I don't know the vaccine	1	0.70	25.35	
I don't trust it	1	0.70	26.06	
I don't trust the West	1	0.70	26.76	
I don't trust the source	1	0.70	27.46	
I don't trust the system	1	0.70	28.17	
I dont have interest in vaccines	1	0.70	28.87	
I dont hink is neccessary if i have a good immunity	1	0.70	29.58	
I dont know how safe is the vaccine	1	0.70	30.28	
I have been exposed and developed immunity already	1	0.70	30.99	
I have never tested positive	1	0.70	31.69	
I have problem with the efficacy	1	0.70	32.39	
I have to be sure of its potency	1	0.70	33.10	
I love to be with my family	1	0.70	33.80	
I need to know  how safe it is	1	0.70	34.51	
I once received a vacin which I think is for trial I nearly died	1	0.70	35.21	
I will build my immune system	1	0.70	35.92	
I will follow all precautionary measures of covid19	1	0.70	36.62	
I will not  vaccine take  so that  tomorrow it's  will turn to another thing	1	0.70	37.32	
I will take the precaution measures	1	0.70	38.03	
I'm not ready for it	1	0.70	38.73	
I'm not sure of it's effectiveness	1	0.70	39.44	
I'm still afraid	1	0.70	40.14	
If only it's safe	1	0.70	40.85	
Immunity is built by what you eat	1	0.70	41.55	
It could be risky	1	0.70	42.25	
It is dangerous	1	0.70	42.96	
It is not a prophylaxis	1	0.70	43.66	
It is not approved yet	1	0.70	44.37	
It may have a long term side effects	1	0.70	45.07	
It's not safe	1	0.70	45.77	
It's unnecessary	1	0.70	46.48	
More death will occur	1	0.70	47.18	
Never tested positive	1	0.70	47.89	
No drug has been specific for Covid-19	1	0.70	48.59	
No much information on the vaccine	1	0.70	49.30	
No need	2	1.41	50.70	
No specific reason	1	0.70	51.41	
No trust in the vaccine	1	0.70	52.11	
No vaccine	1	0.70	52.82	
None	1	0.70	53.52	
Not fully convinced of the source	1	0.70	54.23	
Not immediately it is introduced, would want to wait for awhile before giving it a try	1	0.70	54.93	
Not infected	1	0.70	55.63	
Not interested	3	2.11	57.75	
Not interested	1	0.70	58.45	
Not knowing the effect	1	0.70	59.15	
Not necassary	1	0.70	59.86	
Not necessary	1	0.70	60.56	
Not ready	1	0.70	61.27	
Not reliable	1	0.70	61.97	
Not safe	1	0.70	62.68	
Not safe	2	1.41	64.08	
Not sure	1	0.70	64.79	
Not sure	1	0.70	65.49	
Not sure if it's 100%tested	1	0.70	66.20	
Not sure if it's safe	1	0.70	66.90	
Not sure of it	1	0.70	67.61	
Not sure of it safety	1	0.70	68.31	
Not sure of side effects	1	0.70	69.01	
Nothing	6	4.23	73.24	
Once vaccinated you introduce a percentage of the virus into your human system	1	0.70	73.94	
Personal	1	0.70	74.65	
Personal	2	1.41	76.06	
Protection	1	0.70	76.76	
Safety is not guaranteed	1	0.70	77.46	
Same	1	0.70	78.17	
Same reason	1	0.70	78.87	
Same reason above	1	0.70	79.58	
Same reason.	1	0.70	80.28	
Scared	2	1.41	81.69	
Scared	1	0.70	82.39	
Scared of it	1	0.70	83.10	
Since l was able to recover my immunity is strong	1	0.70	83.80	
Sources can't be trusted and getting immunized is not the e best	1	0.70	84.51	
The disease will go away	1	0.70	85.21	
The medical constituents must be known first	1	0.70	85.92	
There are others ways to boast my immunity	1	0.70	86.62	
There is no approved and recommended vaccine	1	0.70	87.32	
There is no approved vaccine	1	0.70	88.03	
Till I no where the source of the vaccine	1	0.70	88.73	
Try & error on us	1	0.70	89.44	
Unknown side effects	1	0.70	90.14	
Unless I have absolute knowledge on the vaccine and it's implications	1	0.70	90.85	
Unless is tested and confirmed	1	0.70	91.55	
Want to be sure  of the potency of the vaccine	1	0.70	92.25	
We don't whether it is safe or not	1	0.70	92.96	
Would rather go by the natural preventive measures	1	0.70	93.66	
Would wait for d response from those who are willing to take it first	1	0.70	94.37	
because is not yet confirmed	1	0.70	95.07	
confidential	1	0.70	95.77	
if only I'm not contraindicated to the vaccine	1	0.70	96.48	
not in the first batch	1	0.70	97.18	
not necessary	1	0.70	97.89	
not sure of the outcome	1	0.70	98.59	
same as previous answer	1	0.70	99.30	
there is inconsistency in the findings which make not safe for me	1	0.70	100.00	
Total	142	100.00		
	
